# Association between neighborhood food environment and dietary diversity score among older people in Beijing, China: A cross-sectional study

**DOI:** 10.3389/fnut.2022.903214

**Published:** 2022-09-20

**Authors:** Man Zhang, Na Zhang, Mingzhu Zhou, Guansheng Ma

**Affiliations:** ^1^Department of Nutrition and Food Hygiene, School of Public Health, Peking University, Beijing, China; ^2^Laboratory of Toxicological Research and Risk Assessment for Food Safety, Peking University, Beijing, China

**Keywords:** food environment, dietary quality, dietary diversity score, DDS, older adults

## Abstract

**Objective:**

To examine the association between the neighborhood food environment and dietary diversity score (DDS) among elderly people in China.

**Methods:**

Participants were recruited from 12 communities in Beijing, China, in 2019, using a multi-stage stratified random sampling method. Participants (*n* = 1,764, 730 men) in this study were elderly people aged 65 to 80. A questionnaire survey was used to investigate the intake of various foods in the past 3 days, and their dietary diversity score (DDS) was calculated. Baidu Map Application Programming Interface was used to measure the neighborhood food environments, including the density of and proximity to different food outlets. Adjusted multiple linear regression was performed to estimate the association between the neighborhood food environment and DDS.

**Results:**

A total of 1,800 questionnaires were distributed, and 1,775 questionnaires were returned. The questionnaire response rate was 98.6%. Among them, the number of valid questionnaires was 1,764, and the valid rate was 99.4%. The mean age of the participants was 69.7 ± 4.3, and the average DDS was 7.2 ± 1.4. Among the three types of stores, convenience stores had the best access, followed by greengrocers, and finally supermarkets. Sit-down restaurants had the nearest walking distance, Chinese fast-food restaurants had the largest number, and western fast-food restaurants were the most difficult to access. Better access to supermarkets tended to be associated with higher DDS score within all the buffer zones (250 m buffer zone: β = 0.495, *P* < 0.001; 500 m buffer zone: β = 0.341, *P* < 0.001; 800 m buffer zone: β = 0.163, *P* < 0.001; 1,000 m buffer zone: β = 0.243, *P* < 0.001). However, greengrocers were negatively associated with DDS score within all the buffer zones (250 m buffer zone: β = −0.475, *P* < 0.001; 500 m buffer zone: β = −0.161, *P* < 0.001; 800 m buffer zone: β = −0.090, *P* < 0.001; 1000 m buffer zone: β = −0.112, *P* < 0.001). As for convenience stores, we only found significant results within the 250 m buffer zone (β = 0.075, *P* = 0.002). Among the three types of restaurants, the results were inconsistent within different buffer zones. Sit-down restaurants were negatively associated with DDS score within 250 m buffer zone (β = −0.257, *P* < 0.001), and positively associated with DDS score within 1,000 m buffer zone (β = 0.018, *P* < 0.001). Living in areas with more Chinese fast-food restaurants tended to have higher DDS within 250 m buffer zone (β = 0.357, *P* < 0.001); however, there was a opposite result within 1,000 m buffer zone (β = −0.044, *P* < 0.001). Better access to western fast-food restaurants tended to be associated with lower DDS score within 500 m buffer zone (β = −0.235, *P* < 0.001) and higher DDS score within 1,000 m buffer zone (β = 0.189, *P* < 0.001). There was a negative correlation between the nearest distance to the supermarket and the DDS score (β = −0.002, *P* < 0.001), and the nearest distance to the greengrocer was directly positively correlated with the DDS score (β = 0.004, *P* < 0.001).

**Conclusion:**

This study suggests that supermarkets may increase the DDS score among older adults in Beijing, while greengrocers may reduce the DDS score. However, the current results are not strong enough to draw specific conclusions. Policymakers need to rely on more evidence to make specific policy recommendations.

## Introduction

In recent years, the incidence rate of chronic diseases in the world has increased year by year. According to the latest *Report on Chinese residents' chronic diseases and nutrition 2020*, the prevalence of hypertension, diabetes, and hypercholesterolemia among residents aged 18 and above was 27.5, 11.9, and 8.2%, respectively; the prevalence of the chronic obstructive pulmonary disease among residents aged 40 and above was 13.6%, which was all increased compared with the results released in 2015 (1). In 2019, the death rate of Chinese residents from chronic diseases was 685/100,000, accounting for 88.5% of the total death, of which the death rate caused by cardiovascular and cerebrovascular diseases, cancer and chronic respiratory diseases accounted for 80.7% (1).

Unreasonable diet is an important factor in the development of chronic diseases. Dietary diversity means different types of food or foods within the same group intake within a specific period of time (2). Dietary diversity is not only the key factor to improve dietary quality but also the guarantee to promote health. Many studies have shown that the degree of dietary diversity is positively correlated with nutrient intake and its adequacy (3, 4). Dietary diversity can be used to predict nutrient adequacy. The degree of dietary diversity is also related to the occurrence and development of chronic diseases. The level of dietary diversity is negatively correlated with the incidence of metabolic syndrome (5), cardiovascular diseases (6), diabetes (7), and other diseases (3). The risk of hyperlipidemia, hypertension and diabetes decreased with the increase of dietary diversity score (3, 8, 9). The evaluation methods of dietary diversity include simple counting method and comprehensive index method. The simple counting method only counts the types of food without considering the recommended intake of nutrients; the comprehensive index method not only counts the food types, but also considers the recommended intake of nutrients to comprehensively evaluate dietary diversity (3). Simple counting method is represented by dietary diversity score (DDS) and food variety score (FVS), and comprehensive index method is represented by diet quality index (DQI) and health eating index (HEI) (3). Among these methods, DDS has high efficiency in predicting the sufficiency of nutrients. At the same time, it has the characteristics of simplicity and good compliance of survey objects. It is especially suitable for the rural population and the elderly population (3). Therefore, although DDS has some limitations, it was often chosen as the evaluation index of dietary diversity.

Dietary behavior has complex influencing factors, including genetic, socioeconomic, and cultural factors. The food environment has emerged as a key contributor to dietary behavior. The food environment is defined as the collective physical, economic, policy, and sociocultural surroundings, opportunities, and conditions that influence people's food and beverage choices and nutritional status (10). The neighborhood food environment, also known as community food environment, is commonly characterized as the number, type, location, and accessibility of multiple food outlets, such as convenience stores, supermarkets, fast-food restaurants, and the availability, affordability, and acceptability of foods in a community's food outlets (11, 12). In areas with low accessibility, availability, affordability and acceptability of food outlets, there may exists a “food desert”. Food desert refers to low-income geographic area that lacks access to a supermarket or large grocery store (13). The concept of “food desert” has now developed into “low income and low access (LILA) census tracts”. According to Food Access Research Atlas, LILA census tracts are low-income tracts with at least 500 people, or 33 percent of the population, living more than 1 mile (urban areas) or more than 10 miles (rural areas) from the nearest supermarket, supercenter, or large grocery store. Food deserts exist in many countries (14). In 2015, 39.4 million people, or 12.8 percent of the U.S. population, lived in food deserts (14). Food deserts may contribute to social disparities in diet and diet-related health outcomes, such as cardiovascular disease and obesity (15, 16). Eliminating food deserts could improve diet either by providing access to healthy foods for individuals seeking those foods but with prior poor access or by generating demand within communities that had limited exposure to healthy foods (13).

There are many methods to measure the neighborhood food environment, such as questionnaire or phone survey, commercial data list, and Geographic Information System (GIS) Technology (17). The Baidu Map Application Programming Interface (API) (Baidu, Beijing, China) used to be widely used in fields of transportation, surveying, and mapping engineering (18, 19). Baidu Map API has now been introduced into the field of public health and has been proved effective in assessing the food environment (20).

Globally, more and more scholars began to pay attention to the associations between the neighborhood food environment and dietary intake. These studies did not come to a consistent conclusion, which may be due to the diversity of food environmental indicators, the difference of geographical location, and the heterogeneity of the study population (21). However, there were still much meaningful evidence. McInerney et al. found that there was a positive association between the density of food outlets and the dietary diversity, nutrient adequacy, and diet quality among Canadian adults (22). Morland et al. reported that the intake of fruits and vegetables increased by 32% for each additional supermarket in black American living areas (23). Skidmore et al. found that better access to convenience stores was positively associated with high-calorie food intake (24). Different types of restaurants also have different effects on dietary intake. Longacre et al. documented the density of fast-food restaurants to be positively associated with fast-food consumption among American urban adolescents and adults (25). Another study showed that western fast-food restaurants that mainly provided hamburgers, fried chicken and other foods were related to higher energy, fat and sugar intake; Dinner restaurants that offered a wide variety of food were associated with higher vegetable intake (26).

The number of studies on the relationship between the food environment and dietary intake is relatively small in China. A study reviewed the literature on the neighborhood food environment in relation to diet among residents in China, and it found that 6 out of 8 studies reported a significant relationship, whereas two studies reported a null association (21). However, most studies in China used subjective measurement methods such as questionnaire surveys, and this may result in recall bias and social expectation bias (21, 27). At the same time, the influence of the food environment may be highly specific to different populations. For example, school-aged children are influenced by both school and family food environments, and the working-age population is influenced by both worksite and family food environments. However, the dietary intake of older people may be most influenced by their neighborhood food environments (28). China has been an aging society since 1999 (29). At the end of 2020, the population of those over 60-year old in China reached 264 million, and the population of those over 65 years old reached 191 million, accounting for 18.7 and 13.5%, respectively (30), ranking first in all countries in the world (31). Therefore, we should pay more attention to the elderly. Due to the inconsistent research results and the lack of Chinese-based evidence and older people's evidence, more studies on the relationship between the neighborhood food environment and dietary intake are needed.

In this study, we investigated the relationship between neighborhood food environment and DDS among older people in Beijing, China. The objectives of this study are: to clarify the association between different types of food outlets and DDS; to identify the key types of food outlets that affect the DDS of the elderly; and to make recommendations to improve the dietary intake and health of the older adults.

## Materials and methods

### Participants

This study was based on a cross-sectional survey conducted in 2019, which was to examine the dietary behavior and influencing factors of the older people. We recruited participants from 12 communities in Beijing, China. Qualified older adults were invited by phone or door-to-door by researchers and community staff. Overall, 1,800 older people were invited to participate in this survey, and 98.6% responded. Valid questionnaires meant that the participant provided complete information on age, gender, marital status, and food intake information. Finally, 1,764 were valid questionnaires. The efficiency of the questionnaire was 99.4%.

Inclusion criteria: 65~80 years old, retired from work, community-dwelling, living in only one community, living in the current community for more than 2 years, functionally independent.

Exclusion criteria: unable to eat normally; cognitively impaired.

### Sample

#### Sampling method

A multi-stage stratified random sampling method was used, and the specific steps were as follows:

Firstly, three districts that represented different geographic locations and economic levels of Beijing were selected as the target districts: Haidian District, Shunyi District, and Miyun District. Among these three districts, Haidian District has the closest distance to the center of Beijing and highest economic level, followed by Shunyi District and Miyun District;

Secondly, one urban street and one suburban street were selected from each district; Thirdly, two communities were selected from each street; Lastly, qualified older people were randomly selected from each community.

#### Sample size calculation

For the calculation of the sample size, the following formula was used:


$$\begin{array}{l} N\;\;=Z_{1-\alpha /2^{2}}\;p(1-p)/e^{2}. \end{array}$$

Among these parameters, α = 0.05, Z_1−α/2_ = 1.96, e = 0.03; *p* = 0.15 indicated the obesity rate of older people in China (32, 33). Besides, a 10% dropout rate was considered. Three districts were investigated, and the final sample size was 1,800.

### Ethical review

The study protocol was reviewed and approved by the Peking University Biomedical Ethics Committee. The ethical approval project identification code is IRB00001052-17112. The study was carried out according to the principles of the Declaration of Helsinki. All participants read the informed consent form, voluntarily agreed to participate in this study, and signed the informed consent form before the study. Written informed consent was obtained from each participant before enrolment in the study and then preserved by researchers.

### Participants' basic information

The participants' basic information was investigated by questionnaires. Basic information included: address, age, gender, marital status, education level, income level, frequency of exercise, and frequency of smoking and drinking. Basic information was self-reported by participants and filled out one by one by the investigators and the participants. Neighborhood socioeconomic level was determined according to the National Bureau of Statistics' documents (34).

### Participants' food intake information

The participants' food intake information was collected through a questionnaire. The questionnaire has been applied in many previous studies and proved effective (2, 9, 35). In the questionnaire, the participants were asked to answer the question: “Have you eaten this kind of food in the past 3 days?” The answer options were classified as “yes” or “no”. The food classification was based on the balanced diet pagoda of Chinese residents: cereals, vegetables, fruits, animal meat, fish and shrimp, eggs, milk and dairy products, beans and soy products, oil and fat. See Table 1 for details of food items for each food classification. We only investigated whether the participants had eaten these foods, and did not consider the frequency and amount of food intake. Foods other than these 9 categories of food are not included in the survey, such as carbonated beverages, alcoholic beverages, coffee, candy, etc. The information collected in this questionnaire would be used to calculate the DDS.

**Table 1:** Food classification applied in DDS.

**Food classification**	**Food items**
Cereals	Cereals and their products
Vegetables	Various vegetables: leafy vegetables, fresh beans, rhizomes, melons, eggplant fruits, bacteria and algae, etc.
Fruits	Fresh fruit and dried fruit
Animal meat	Animal and poultry meat such as pork, chicken, beef
Fish and shrimp	Fish, shrimp, shellfish
Eggs	Fresh and salted eggs of various animals
Beans and soy products	Soybean and its products such as soymilk and tofu
Milk and dairy products	Milk, yogurt and other dairy products
Oil and fat	Animal oil, vegetable oil

The questionnaire was filled in by the investigator and the participants one-on-one. After filling in, the quality control personnel checked each questionnaire. If there was any wrong filling or omission, the respondents supplemented immediately.

### Neighborhood food environment measurement

#### Usage of Baidu map API

Baidu Map API was used to measure the neighborhood food environment, including names and coordinates of food outlets around the community (36). The process of using the Baidu Map API was as follows (20): (1) Call the Geocoding API, and enter the community address to obtain the communities' coordinates; (2) Call the Place API, use the circular area search method, enter the community coordinates and POI (point of interest) keywords, such as supermarkets, stores, convivence stores, restaurants, etc., to obtain the POI names and coordinates within the target radius (1,000 m) around the community; (3) Call the Direction API, use the walking query retrieval service, enter the coordinates of the community and its surroundings to obtain the walking distance. (4) Filter the POI information within the following target walking distance: 250, 500, 800, and 1,000 m. These distances were determined on the following evidence (37, 38): the most frequent space for older people in daily life was within 250 m from home, which was about 5 min walking distance; the main shopping area for older people was within 500 m from home, which was about 10 min walking distance; only about 10% of older people would buy food beyond 1,000 m from home. After these steps, a list of food outlets was obtained.

#### Classification of food outlets

The food outlets were classified into different types according to the National Economic Industry Classification (GB T 4754-2017) (39), Tian Yancha (40), and Dianping (41), as well as the investigators' knowledge of the local area food chains. Based on National Economic Industry Classification, the definition of different types of food establishments was determined. Afterward, all food outlets were divided into different types by checking their business scope in Tian Yancha and Dianping. Tian Yancha is a website for querying corporate information such as industrial and commercial information. Tian Yancha data come from the National Enterprise Credit Information Disclosure System, China Judgment Documents Network, China Enforcement Information Open Network, State Intellectual Property Office, Trademark Office, Copyright Office, and other authoritative websites. So, the information from Tian Yancha is reliable and accurate. Dianping is a website offering local business search, user-generated reviews, detailed business information, and other merchant services.

The list of food outlets mentioned previously was refined to the following categories: (1) supermarket: comprehensive retail activities in supermarkets that sell fresh food, processed food, pre-packaged food, and daily necessities, etc.; (2) convenience store: retail activities in the form of small supermarkets to meet the proportional needs of customers as the main purpose, such as convenience stores and small grocery stores with limited selection; (3) greengrocer: retail activities specializing in fresh vegetables and fruits; (4) sit-down restaurant: catering activities that provide a variety of dishes for lunch and dinner in a certain place, and deliver meals by the waiter, such as Chinese dinners, western dinners, hot pots, etc.; (5) Chinese fast-food restaurant: fast and convenient Chinese catering services in a certain place or through specific equipment, such as Chinese set meals, fried rice, noodles, steamed buns, local cuisines, etc.; (6) western fast-food restaurant: fast and convenient western catering services in a certain place or through specific equipment, such as McDonalds and Kentucky Fried Chicken, pizzerias, limited-service facilities, such as Subway, etc. . Some food outlets in the neighborhoods were excluded from the study because they did not fit the classifications used, such as dessert shops, coffee shops, bars, etc.

### Variables

#### DDS

DDS was the outcome variable of this paper. In part 2.5, we described the questionnaire to collect food intake information. For the 9 types of food we investigated, if the answer was “yes”, 1 point would be given; if the answer was “no”, 0 point would be given. Add the scores of the 9 food categories to get the total score of DDS. The minimum score was 0 and the maximum score was 9.

#### Neighborhood food environment variables

In this study, the density of the food outlets meant the number of food outlets within different walking distances. There were six types of food outlets (supermarkets, convenience stores, greengrocers, sit-down restaurants, Chinese fast-food restaurants, western fast-food restaurants) and four walking distance buffer zones (250, 500, 800, and 1,000 m), so we had 24 food environment variables in total. If a store was both a greengrocer and a convenience store, it was categorized as both a convenience store and a greengrocer.

Proximity to food outlets meant the nearest distance to the different types of food outlets. If no food outlet was found within 1,000 m walking distance, then we searched in a larger walking distance.

#### Confounders

Confounders were as follows: individual-level sociodemographic characteristics including age, gender, marital status, education level, income level; behavioral factors including frequency of exercise, frequency of smoking and drinking; the communities' economic level. These confounders were determined after literature review (42) and expert discussions.

### Statistical analysis

Statistical analyses were performed using SPSS Statistics 20.0 (IBM Corp., Armonk, NY, USA). Participant and food environment characteristics were examined with descriptive statistics. The characteristics of the participants were expressed in frequency and percentage. Participants' neighborhood food environment characteristics were expressed by median and inter-quartile range. Multiple linear regression analysis was performed on the association between DDS and each food environment variable. In Model 1, only the food environment variables were included. To adjust for potential confounders, demographic characteristics (age, gender, marital status, education level, income level), neighborhood socioeconomic level (urban or suburban), and behavioral factors (drinking, smoking, and frequency of exercise) were included in Model 2. *P* < 0.05 was considered statistically significant.

## Results

### Participants characteristics

Participants (*n* = 1,764) had a mean age of 69.7 ± 4.32 years and an average DDS of 7.2 ± 1.4. Over 60% of the elderly were overweight and obese. The sex ratio of men and women was about 1/1.4. The number of older people from urban and suburban areas was approximately the same. Only 17.0% of the elderly had a habit of smoking, and 75.1% drank less than once a week. More details of individual characteristics are shown in Table 2.

**Table 2:** Characteristics of the participants (*n*, %).

**Items**	***n***	**%**
BMI (kg/m^2^)^a^		
Underweight: <18.5	7	0.4
Normal: 18.5–24.9	638	36.2
Overweight: 25–29.9	620	35.1
Obese: ≥30	499	28.3
Gender		
Male	730	41.4
Female	1,034	58.6
Age		
65–69	983	55.7
70–74	483	27.4
75–79	298	16.9
Marital status		
Unmarried	3	0.2
Married	1,482	84.0
Widowed	260	14.7
Divorced or separated	19	1.1
Education level		
≥Bachelor's degree	190	10.8
Middle school^b^	923	52.3
≤ Primary school	651	36.9
Income level (RMB)^c^		
≤ 2,000	409	23.2
2,000–3,500	652	37.0
3,500–5,000	376	21.3
5,000–10,000	223	12.6
≥10,000	24	1.4
Missing	80	4.5
Neighborhood socioeconomic level		
Urban	899	51.0
Suburban	865	49.0
Frequency of exercise		
Never	198	11.2
1–2/week	55	3.1
3–4/week	83	4.7
5–6/week	35	2.0
Everyday	1,387	78.6
Missing	6	0.3
Smoking^d^		
No	1,464	83.0
Yes	300	17.0
Drinking		
≥Once a week	436	24.7
< Once a week	1,325	75.1
Missing	3	0.2

a Body mass index: a measurement of someone's weight in relation to their height

b Including high school, junior high school, technical secondary school, professional technical school, etc.

c Monthly household per capita income in renminbi (RMB): ≤ 2,000 (low level), 2,000–3,500 (low to medium level), 3,500–5,000 (medium level), 5,000–10,000 (medium to high level), ≥10,000 (high level).

d No: never smoked or has quit smoking; Yes: still smoking or has failed to quit smoking.

### Neighborhood food environment characteristics

Among the three types of stores, convenience stores had the most number and nearest distance, followed by greengrocers, and finally supermarkets. Among these three types of restaurants, Chinese fast-food restaurants had the most number, sit-down restaurants had the nearest distance, and western fast-food restaurants had the least number and farthest distance. Among all types of food outlets, western fast-food restaurants had the worst access, whose nearest distance of two communities even reached more than 8,000 m. Detailed information on participants' neighborhood food environment density/proximity characteristics is shown in Tables 3, 4.

**Table 3:** Participants' neighborhood food environment density characteristics.

**Numbers of food outlets**	**250 m buffer zone**		**500 m buffer zone**		**800 m buffer zone**		**1,000 m buffer zone**	
	**Median**	**IQR**	**Median**	**IQR**	**Median**	**IQR**	**Median**	**IQR**
Number of supermarkets	0.00	0.75	0.00	1.00	1.00	1.00	1.50	2.00
Number of convenience stores	1.00	2.75	1.00	5.75	3.50	8.75	5.50	11.50
Number of greengrocers	0.00	1.00	1.00	2.00	3.00	2.50	3.00	3.75
Number of sit-down restaurants	0.00	1.00	1.50	3.75	5.00	10.00	7.00	17.25
Number of Chinese fast-food restaurants	0.00	1.00	1.50	5.00	7.00	14.75	10.00	21.25
Number of western fast-food restaurants	0.00	0.00	0.00	0.75	0.00	2.00	1.00	3.75

IQR, inter-quartile range.

**Table 4:** Participants' neighborhood food environment proximity characteristics.

**Nearest distance to food outlets (m)**	**Median**	**IQR**	**Minimum**	**Maximum**
Nearest distance to supermarkets (m)	557.00	505.00	91.00	1,676.00
Nearest distance to convenience stores (m)	207.50	358.50	0.10	825.00
Nearest distance to greengrocers (m)	304.00	422.50	77.00	705.00
Nearest distance to sit-down restaurants (m)	325.00	299.25	52.00	992.00
Nearest distance to Chinese fast-food restaurants (m)	389.50	520.50	169.00	3,563.00
Nearest distance to western fast-food restaurants (m)	902.50	1,113.25	339.00	8,332.00

The distance is from the food outlet to the neighborhood. IQR, inter-quartile range.

### Association between neighborhood food environment and DDS

Table 5 shows the results of the regression analyses of neighborhood food environment density characteristics and DDS for Model 1 and Model 2. In total, 23 food environment variables (five/six types of stores and four buffer zones) were analyzed for their possible association with DDS. Among the three types of stores, we found that better access to supermarkets tended to be associated with higher DDS score within all the buffer zones (250 m buffer zone: β = 0.495, *P* < 0.001; 500 m buffer zone: β = 0.341, *P* < 0.001; 800 m buffer zone: β = 0.163, *P* < 0.001; 1,000 m buffer zone: β = 0.243, *P* < 0.001), after controlling for potential confounders. However, greengrocers were negatively associated with DDS score within all the buffer zones (250 m buffer zone: β = −0.475, *P* < 0.001; 500 m buffer zone: β = −0.161, *P* < 0.001; 800 m buffer zone: β = −0.090, *P* < 0.001; 1,000 m buffer zone: β = −0.112, *P* < 0.001), after controlling for potential confounders. As for convenience stores, we only found significant results within the 250 m buffer zone: those who live in areas with more convenience stores tended to have higher DDS, one additional convenience store in the neighborhood was associated with a 0.075-point increase in DDS score (*P* = 0.002), after controlling for potential confounders. Among the three types of restaurants, the results were inconsistent within different buffer zones. Sit-down restaurants were negatively associated with DDS score within 250 m buffer zone (β = −0.257, *P* < 0.001), and positively associated with DDS score within 1,000 m buffer zone (β = 0.018, *P* < 0.001), after controlling for potential confounders. Living in areas with more Chinese fast-food restaurants tended to have higher DDS within 250 m buffer zone (β = 0.357, *P* < 0.001); however, there was a opposite result within 1,000 m buffer zone, those living with more Chinese fast-food restaurants tended to have lower DDS score (β = −0.044, *P* < 0.001), after controlling for potential confounders. Better access to western fast-food restaurants tended to be associated with lower DDS score within 500 m buffer zone (β = −0.235, *P* < 0.001). By contraries, within 1,000 m buffer zone, living with more western fast-food restaurants tended to have higher DDS score (β = 0.189, *P* < 0.001). No direct associations were found between other food environment variables and DDS scores after controlling for potential confounders.

**Table 5:** Associations between the density of food outlets and DDS.

**Number of food outlets within different buffer zones**	**Model 1^a^**			**Model 2^b^**		
	**β**	**SE**	***P***	**β**	**SE**	***P***
**Within 250 m buffer zone**						
Number of supermarkets	0.847	0.114	<0.001	0.495	0.111	<0.001
Number of convenience stores	0.140	0.026	<0.001	0.075	0.024	0.002
Number of greengrocers	−0.738	0.050	<0.001	−0.475	0.052	<0.001
Number of sit-down restaurants	−0.359	0.045	<0.001	−0.257	0.045	<0.001
Number of Chinese fast-food restaurants	0.702	0.070	<0.001	0.357	0.069	<0.001
**Within 500 m buffer zone**						
Number of supermarkets	0.100	0.064	0.121	0.341	0.083	<0.001
Number of convenience stores	0.063	0.016	<0.001	0.075	0.055	0.173
Number of greengrocers	−0.179	0.024	<0.001	−0.161	0.022	<0.001
Number of sit-down restaurants	−0.041	0.032	<0.001	−0.026	0.031	0.400
Number of Chinese fast-food restaurants	0.234	0.017	<0.001	0.002	0.054	0.971
Number of western fast-food restaurants	−0.453	0.056	<0.001	−0.235	0.046	<0.001
**Within 800 m buffer zone**						
Number of supermarkets	0.965	0.071	<0.001	0.163	0.042	<0.001
Number of convenience stores	−0.052	0.015	0.001	0.025	0.072	0.729
Number of greengrocers	−0.140	0.024	<0.001	−0.090	0.014	<0.001
Number of sit-down restaurants	0.072	0.007	<0.001	0.049	0.027	0.065
Number of Chinese fast-food restaurants	−0.233	0.023	<0.001	−0.008	0.041	0.846
Number of western fast-food restaurants	0.829	0.066	<0.001	0.037	0.038	0.330
**Within 1,000 m buffer zone**						
Number of supermarkets	0.621	0.079	<0.001	0.243	0.052	<0.001
Number of convenience stores	−0.074	0.021	<0.001	0.051	0.152	0.738
Number of greengrocers	−0.091	0.025	<0.001	−0.112	0.020	<0.001
Number of sit-down restaurants	0.028	0.005	<0.001	0.018	0.004	<0.001
Number of Chinese fast-food restaurants	−0.089	0.015	<0.001	−0.044	0.013	<0.001
Number of western fast-food restaurants	0.487	0.062	<0.001	0.189	0.050	<0.001

SE, standard error.

a Non-adjusted model.

b Adjusted for age, gender, marital status, educational attainment, income level, neighborhood socioeconomic level, drinking, smoking, and frequency of exercise. Results are presented without associated covariate information for conciseness.

Table 6 shows the results of the regression analyses of neighborhood food environment proximity characteristics and DDS score for Model 1 and Model 2. In total, six proximity variables were analyzed for their possible association with DDS score. After controlling for potential confounders, there was a negative association between the nearest distance to supermarkets and the DDS score (β = −0.002, *P* < 0.001), and the nearest distance to the greengrocer was directly positively correlated with the DDS score (β = 0.004, *P* < 0.001). That is, better access to supermarkets was associated with higher DDS scores, and better access to greengrocers was associated with lower DDS scores. No direct associations were found between other food environment proximity variables and DDS score after controlling for potential confounders.

**Table 6:** Associations between proximity to food outlets and DDS.

**Nearest distance to food outlets**	**Model 1^a^**			**Model 2^b^**		
	**β**	**SE**	***P***	**β**	**SE**	***P***
Nearest distance to supermarkets	−0.002	0.000	<0.001	−0.002	0.000	<0.001
Nearest distance to convenience stores	0.000	0.000	0.016	0.046	0.036	0.534
Nearest distance to greengrocers	0.004	0.001	<0.001	0.004	0.001	<0.001
Nearest distance to sit-down restaurants	−0.001	0.040	0.857	−0.011	0.028	0.854
Nearest distance to Chinese fast-food restaurants	−0.001	0.000	0.002	−0.039	0.033	0.635
Nearest distance to western fast-food restaurants	−0.025	0.032	0.747	−0.006	0.034	0.672

SE, standard error.

a Non-adjusted model.

b Adjusted for age, gender, marital status, education level, income level, neighborhood socioeconomic level, drinking, drinking, smoking, and frequency of exercise. Results are presented without associated covariate information for conciseness.

## Discussion

Our results showed that in 250, 500, 800, and 1,000 m buffer zone, the number of supermarkets was positively associated with DDS score, while the number of greengrocers was associated with lower DDS score among older adults in Beijing, China. At the same time, we found that there was a negative association between the nearest distance to supermarkets and DDS score, and a positive association between the nearest distance to greengrocers and DDS score. In other words, better access to supermarkets was associated with higher DDS score, while better access to greengrocers was associated with lower DDS score. This may be because, compared with other types of food outlets, supermarkets provide a wide range of foods, including fresh vegetables and fruits, dairy products, fish, poultry, meat and eggs, cereals and potatoes, nuts, etc. (43, 44). On the contrary, greengrocers mainly sell fruits and vegetables and less sell other types of food. When the accessibility of the greengrocers in the neighborhood was high, the elderly often went to greengrocers to buy food, which may reduce the consumption of other food, thus reducing the DDS score. However, this did not mean that greengrocers are not conducive to healthy diet. The high accessibility of greengrocers may increase the intake of vegetables and fruits, which was good to health. Therefore, if we chose some other dietary indicators that pay special attention to the intake of vegetables and fruits, we may get different results. The current study also showed that, the construction of large supermarkets around the neighborhood may improve the dietary diversity of the elderly. However, our previously published research found that supermarkets may be related to the high BMI of the elderly (17). Therefore, it is important to improve the consumption environment inside the supermarkets and educate the elderly to choose healthy food.

The greater the number of convenience stores within a 250 m buffer zone, the higher the DDS of the elderly. Although the previous studies reported proximity to convenience stores to be positively associated with high-calorie food intake (24), and our previously published research found a positive association between convenience stores accessibility and BMI (17), convenience stores may be beneficial to improving dietary diversity for the elderly. This may be because when we used DDS to evaluate the dietary quality, we only divided the food into nine categories and considered the type of food, but we did not consider other aspects of food, such as the amount of food, cooking methods, the amount of oil, salt and sugar added, etc. These factors will also significantly affect the quality of diet, thus affecting the BMI and health status of the elderly.

We also found significant associations between different restaurants and DDS, but the results were inconsistent, with associations for the same type of relationship being positive, negative, or non-existent in the different buffer zone. This may be due to the complexity of food sold in different restaurants and the diversity of Chinese food culture. For example, Chinese fast-food restaurants have a wide variety of categories, and there are obvious differences among these categories (45, 46). The food sold in different restaurants is very different, including steamed stuffed buns, dumplings, noodles, rice noodles, rice, boxed rice, Mala Tang, fried food, and so on (45, 46). Typical fast-food restaurants in China include “real kung fu” which mainly sells meat and vegetable set meals, “Daniang Dumplings” which mainly sells dumplings, “Lanzhou Ramen” which mainly sells noodles, “Yang Guofu Malatang” and “Zhang Liang Malatang” which mainly sell Malatang, “Hongzhuangyuan” and “Jiahe Yipin” which mainly sell porridge. When they choose different restaurants, the types of food they eat will also be different, which will lead to different results of DDS score.

Our study adds evidence to the potential influence of the neighborhood food environment in China. Although we found some significant results in this study, we cannot conclude if a certain type of food outlet is healthy or not. This study only considered the density of and proximity to food outlets in the neighborhood. Other dimensions of the neighborhood food environment were not considered. For example, the affordability, acceptability, accommodation, and the consumption environment of food outlets were not considered (47). In addition, there are individual differences in the way people interact with the food environment (48). There are many influence factors that could mediate or moderate the relationship between the food environment and DDS, such as personal preferences, family food rules, and consumption environments in food outlets. For policymakers, the current evidence is insufficient to make precise recommendations. Relevant research is needed on different dimensions of the food environment. In particular, prospective experimental research is needed to draw causal conclusions.

Our study has a number of strengths, including innovation concerns, exposure and outcome concerns, study design concerns, and statistical analysis concerns (49). Innovation concerns: (1) As far as we know, this was the first study to examine such associations among Chinese older people. Our findings provide an evidence base to the association between the food environment and DDS among older people in China. Our findings can also provide scientific backing for Chinese policymakers. (2) In this study, the new method of Baidu Map API was used to measure the neighborhood food environment. This may provide new ideas for the methodology of future research. Previous methods of measuring the food environment have certain disadvantages. For example, data from commercial lists or provided by government agencies has been questioned for its accuracy (50, 51); the GIS method has the disadvantage of being a complex process and high-cost, and the GIS method is facing challenges about data quality and geolocation accuracy (52). However, Baidu Map API has advantages in the following aspects: convenient development, low investment cost, and good performance (20). Baidu Map API, which has been widely used in transportation and surveying engineering fields, has been proved effective in the study of food environment: the positive predictive values for fast-food restaurants and convenience stores were 0.84 and 0.86, respectively (20). Exposure and outcome concerns: (3) We selected DDS, an indicator of food diversity, as the outcome variable. This was more representative of the dietary quality of the elderly than a single food intake. (4) Our assessment of the neighborhood environment was based on walking distance, not circular buffers. Walking distance showed the actual distances that people must travel to access food. This was more accurate than circular buffers because even a small circular buffer (0.25 miles or 400 m) may include food establishments outside walking distance. Design concerns: (5) No restrictions on the diseased/obese participants to prevent selection bias, and (6) a multi-stage stratified random sampling method was used to solve neighborhood self-selection bias. At the same time, this sampling method ensured that the results could basically represent the overall situation of Beijing. Analysis concerns: (7) There were many confounding factors included in our model, including demographic characteristics (age, gender, marital status, education level, and income level), behavior characteristics (drinking, smoking, and exercise), and neighborhood socioeconomic level.

Our study is subject to a few limitations. First, since the participates of this study were the elderly, we chose DDS, which was easy to implement, as the outcome variable to simply evaluate the dietary quality. However, DDS has some limitations. On the one hand, it does not consider the frequency and amount of food. In fact, the contribution of those foods with low intake to the total score of DDS should be less than those with similar nutritional composition but high consumption. On the other hand, the DDS used in this study divided the food into 9 types, but the food items in the same type may have different nutritional values. For example, fruits include fresh fruit and dry fruit, and their vitamin C content was very different. Second, our study area was limited to Beijing. Due to the high economic level in Beijing, it did not represent the whole situation of China. Our findings need to be replicated in cities of different economic levels in China. Third, food outlet data were not validated in person. Finally, this was a cross-sectional survey, so causal inference cannot be made.

## Conclusions

This study suggests that supermarkets may increase the DDS score among older adults in Beijing, while greengrocers may reduce the DDS score. However, the current results are not strong enough to draw specific conclusions. Policymakers need to rely on more evidence to make specific policy recommendations. In future studies, a bigger and more complete picture of food environments should be considered. It is necessary to carry out relevant research on different dimensions of the food environment, especially prospective experimental research, to draw causal conclusions.

## Data availability statement

The raw data supporting the conclusions of this article will be made available by the authors, without undue reservation.

## Ethics statement

The studies involving human participants were reviewed and approved by Peking University Biomedical Ethics Committee. The patients/participants provided their written informed consent to participate in this study.

## Author contributions

MZha was responsible for the design and implementation of the project, data cleaning, statistical analysis, and completed the first draft of the paper. NZ and MZho participated in the questionnaire investigation and revision of the article. GM was responsible for the on-site quality control of the project and participated in the research design. All authors contributed to the article and approved the submitted version.

## Conflict of interest

The authors declare that the research was conducted in the absence of any commercial or financial relationships that could be construed as a potential conflict of interest.

## Publisher's note

All claims expressed in this article are solely those of the authors and do not necessarily represent those of their affiliated organizations, or those of the publisher, the editors and the reviewers. Any product that may be evaluated in this article, or claim that may be made by its manufacturer, is not guaranteed or endorsed by the publisher.

## References

[1] Disease Control Bureau. National Health Commission. Report on Chinese residents' chronic diseases and nutrition. Beijing: People's Medical Publishing House. (2020).

[2] Jin Y. Study on associations of dietary diversity with nutrients adequacy and nutrition related chronic disease in Chinese adults. In: China Centre for Disease Control and Prevention. (2009). Available online at: http://cdmd.cnki.com.cn/Article/CDMD-84501-1011211180.htm

[3] Shen S Lv XH. Research Progress on the relationship between dietary diversity and health. J North Sichuan Med Coll. (2017) 32:4. 10.3969/j.issn.1005-3697.2017.03.044

[4] Salehi-Abargouei A Akbari F Bellissimo N Azadbakht L. Dietary diversity score and obesity: a systematic review and meta-analysis of observational studies. Eur J Clin Nutr. (2016) 70:1–9. 10.1038/ejcn.2015.118 26220567

[5] Vadiveloo M Parkeh N Mattei J. Greater healthful food variety as measured by the US healthy food diversity index is associated with lower odds of metabolic syndrome and its components in US adults. J Nutrition. (2015) 145:564–71. 10.3945/jn.114.199125 25733473 PMC4336534

[6] Huffman FG Zarini GG McNamara E Nagarajan A. The healthy eating index and the alternate healthy eating index as predictors of 10-year CHD risk in Cuban Americans with and without type 2 diabetes. Public Health Nutr. (2011) 14:2006–14. 10.1017/S1368980011001054 21729463

[7] Tiew KF Chan YM Lye MS Loke SC. Factors associated with dietary diversity score among individuals with type 2 diabetes mellitus. J Health Popul Nutr. (2014) 32:665–76. 25895200 PMC4438697

[8] Azadbakht L Mirmiran P Azizi F. Variety scores of food groups contribute to the specific nutrient adequacy in Tehranian men. Eur J Clin Nutr. (2005) 59:1233–40. 10.1038/sj.ejcn.1602234 16015253

[9] Hu QQ Han XX Ma AG Li XL. Investigation and analysis of dietary diversity score and health status of middle-aged and elderly people in rural areas. Food Nutr China. (2009) 12:58–61. 10.3969/j.issn.1006-9577.2009.12.020

[10] Swinburn B Vandevijvere S Kraak V Sacks G Snowdon W Hawkes C. Monitoring and benchmarking government policies and actions to improve the healthiness of food environments: a proposed. Government Healthy Food Environment Policy Index. Obesity Rev. (2013) 14:24–37. 10.1111/obr.12073 24074208

[11] Story M Kaphingst KM Robinson-O'Brien R Glanz K. Creating healthy food and eating environments: policy and environmental approaches. Ann Rev Public Health. (2008) 29:253–72. 10.1146/annurev.publhealth.29.020907.090926 18031223

[12] Glanz K Sallis JF Saelens BE Frank LD. Healthy nutrition environments: concepts and measures. Am J Health Promot. (2005) 19:330–3. 10.4278/0890-1171-19.5.330 15895534

[13] Block JP Subramanian SV. Moving beyond “food deserts”: reorienting united states policies to reduce disparities in diet quality. PLoS Med. (2015) 12:e1001914. 10.1371/journal.pmed.1001914 26645285 PMC4672916

[14] Economic Research Service, U.S. Department of agriculture (USDA). Food access Research Atlas. Available online at: https://www.ers.usda.gov/data-products/food-access-research-atlas/ (accessed December 20, 2020).

[15] Beaulac J Kristjansson E Cummins S. A systematic review of food deserts, 1966-2007. Prevent Chron Dis. (2009) 6:A105. 10.1177/1466424008094803 19527577 PMC2722409

[16] Walker RE Keane CR Burke JG. Disparities and access to healthy food in the United States: A review of food deserts literature. Health Place. (2010) 16:876–84. 10.1016/j.healthplace.2010.04.013 20462784

[17] Zhang M Guo W Zhang N He HR Zhang Y Zhou MZ. Association between neighborhood food environment and body mass index among older adults in Beijing, China: A cross-sectional study. Int J Environ Res Public Health. (2020) 17:7658. 10.3390/ijerph17207658 33092232 PMC7589694

[18] Li XF Huang HN Li JH Xu H. A research on rail traffic generation forecasting based on Baidu Map API. Shandong Sci. (2017) 30:82–8. 10.3976/j.issn.1002-4026.2017.01.013

[19] Du CM. Application of BaiDu Map API in small geographic information system. Geomat Spat Inf Technol. (2011) 34:152–3. 10.3969/j.issn.1672-5867.2011.02.052

[20] Yang SY Xie RS Deng YS Zong YN Liu L Gao YH. Application of Baidu map API for the study on obesogenic food environment among middle school students. Chinese J School Health. (2018) 39:990–2. 10.16835/j.cnki.1000-9817.2018.07.009

[21] An R He L Shen MJ. Impact of neighbourhood food environment on diet and obesity in China: A systematic review. Public Health Nutr. (2019) 23:1–17. 10.1017/S1368980019002167 31511114 PMC10200541

[22] McInerney M Csizmadi I Friedenreich CM Uribe FA Nettel-Aguirre A McLaren L. Associations between the neighbourhood food environment, neighbourhood socioeconomic status, and diet quality: An observational study. BMC Public Health. (2016) 16:984. 10.1186/s12889-016-3631-7 27633380 PMC5025628

[23] Morland K Wing S Roux AD. The contextual effect of the local food environment on residents' diets: The atherosclerosis risk in communities study. Am J Public Health. (2002) 92:1761–768. 10.2105/ajph.92.11.1761 12406805 PMC1447325

[24] Skidmore P Welch A Sluijs EV Jones A Harvey I Harrison F. Impact of neighbourhood food environment on food consumption in children aged 9–10 years in the UK SPEEDY (Sport, Physical Activity and Eating behaviour: Environmental Determinants in Young people) study. Public Health Nutr. (2010) 13:1022–30. 10.1017/S1368980009992035 20082745 PMC3164802

[25] Longacre MR Drake KM MacKenzie TA Gibson L Owens P Titus LJ. Fast-food environments and family fast-food intake in nonmetropolitan areas. Am J Prev Med. (2012) 42:579–87. 10.1016/j.amepre.2012.02.017 22608373 PMC3369570

[26] Patel O Shahulhameed S Shivashankar R Tayyab M Rahman A Prabhakaran D. Association between full service and fast food restaurant density, dietary intake and overweight/obesity among adults in Delhi, India. BMC Public Health. (2018) 18:36. 10.1186/s12889-017-4598-8 28724371 PMC5518129

[27] Shen J He L An RP. Food environment and its relation to diet behavior and obesity in China. Chin. J Epidemiol. (2019) 40:1296–303. 10.3760/cma.j.issn.0254-6450.2019.10.023 31658534

[28] Yen IH Michael YL Perdue L. Neighborhood environment in studies of health of older adults: a systematic review. Am J Prev Med. (2009) 37:455–63. 10.1016/j.amepre.2009.06.022 19840702 PMC2785463

[29] Chen B. The trend of population aging in China and its influence. China Venture Capital. (2016) 18:13–16. 10.3969/j.issn.1673-5811.2016.18.004

[30] National Bureau of Statistics. Bulletin of the Seventh National Census [EB/OL]. Available online at: https://baike.baidu.com/item/第七次全国人口普查公报/56965952 (accessed May 26, 2021).

[31] World Bank. World Bank staff estimates using the World Bank's total population and age/sex distributions of the United Nations Population Division's World Population Prospects. (2019) Revision [EB/OL].

[32] Song MN Cheng X Kong JX Wang HM. Prevalence and influencing factors of overweight and obesity among middle-aged and elderly people in China. Chin J Dis Control Prev. (2018) 22:804–8. 10.16462/j.cnki.zhjbkz.2018.08.010

[33] Zhang M Jiang Y Li YC Zhao W. Prevalence of overweight and obesity among Chinese elderly aged 60 and above in 2010. Chinese J Epidemiol. (2014) 35:365–9. 10.3760/cma.j.issn.0254-6450.2014.04.005 25009021

[34] National Bureau of Statistics. Announcement on the Update of the National Statistical Division Codes and Urban-Rural Division Codes. Available online at: http://www.stats.gov.cn/tjsj/tjbz/tjyqhdmhcxhfdm/2019/index.html (accessed October 02, 2020).

[35] Jin Y Li YP Hu XQ Cui ZH He YN Ma GS. Association between dietary diversity and nutrients adequacy in Chinese adults. Acta Nutrimenta Sinica. (2009) 31:21–5. 10.3321/j.issn:0512-7955.2009.01.005

[36] Baidu Map Open Platform. Available online: https://lbsyun.baidu.com

[37] Cai H. Space and Environment of Chinese Urban Elderly Communities. Architect. (2003) 04:21–7.

[38] Chai YW Li CX. The spatial characteristics of shopping behavior of the Chinese urban elderly: A case study of Beijing, Shenzhen, and Shanghai. Acta Geogr Sin. (2005) 60:401–8. 10.3321/j.issn:0375-5444.2005.03.006

[39] General Administration of Quality Supervision. China National Standardization Management Committee. Industrial Classification for National Economic Activities. GB T 4754-2017; 2017, Beijing, China: China Statistics Press.

[40] Tian Yancha. Available online at: https://www.tianyancha.com/?jsid=SEM-BAIDU-PZ0824-SY-000001 (accessed November 25, 2019).

[41] Dianping. Available online at: https://www.dianping.com (accessed November 25, 2019).

[42] Hanibuchi T Kondo K Nakaya T Nakade M Kawachi I. Neighborhood food environment and body mass index among Japanese older adults: Results from the Aichi Gerontological Evaluation Study (AGES). Int J Health Geogr. (2011) 10:43. 10.1186/1476-072X-10-43 21777439 PMC3150234

[43] Laraia BA Siega-Riz AM Kaufman JS Jones SJ. Proximity of supermarkets is positively associated with diet quality index for pregnancy. Preventive Medicine. (2004) 39:869–75. 10.1016/j.ypmed.2004.03.018 15475018

[44] Rahkovsky I Snyder S. Food choices and store proximity. Economic Research Report. (2015).

[45] Qiu B. Prospect analysis of four trump categories of Chinese catering. Business Observ. (2021) 24:10–14. Available online at: https://d.wanfangdata.com.cn/periodical/shangygc202124003

[46] Zhang HY Zhang CY. Analysis on the advantages and disadvantages of Chinese fast food and Western fast food. Sci Technol Ecn Market. (2006) 7:184–5. 10.3969/j.issn.1009-3788.2006.07.179

[47] Caspi CE Sorensen G Subramanian SV Kawachi I. The local food environment and diet: A systematic review. Health Place. (2012) 18:1172–87. 10.1016/j.healthplace.2012.05.006 22717379 PMC3684395

[48] Martine S Daniel L Smith NR Christelle C Steven C. Associations between home and school neighbourhood food environments and adolescents' fast-food and sugar-sweetened beverage intakes: findings from the Olympic Regeneration in East London (ORiEL) Study. Public Health Nutr. (2018) 21:2842–51. 10.1017/S1368980018001477 29962364 PMC10260913

[49] Cobb LK Appel LJ Franco M Jones-Smith JC Nur A Anderson CAM. The relationship of the local food environment with obesity: A systematic review of methods, study quality, and results. Obesity. (2015) 23:1331–44. 10.1002/oby.21118 26096983 PMC4482774

[50] Kelly B Flood VM Yeatman H. Measuring local food environments: An overview of available methods and measures. Health Place. (2011) 17:1284–93. 10.1016/j.healthplace.2011.08.014 21908229

[51] Powell LM Han E Zenk SN Khan T Quinn CM Gibbs KP. Field validation of secondary commercial data sources on the retail food outlet environment in the US. Health & Place. (2011) 17:1122–31. 10.1016/j.healthplace.2011.05.010 21741875

[52] Charreire H Casey R Salze P Simon C Chaix B Banos A. Measuring the food environment using geographical information systems: a methodological review. Public Health Nutr. (2010) 13:1773–85. 10.1017/S1368980010000753 20409354

